# SHIFT: speedy histological-to-immunofluorescent translation of a tumor signature enabled by deep learning

**DOI:** 10.1038/s41598-020-74500-3

**Published:** 2020-10-15

**Authors:** Erik A. Burlingame, Mary McDonnell, Geoffrey F. Schau, Guillaume Thibault, Christian Lanciault, Terry Morgan, Brett E. Johnson, Christopher Corless, Joe W. Gray, Young Hwan Chang

**Affiliations:** 1 Computational Biology Program, Department of Biomedical Engineering, Oregon Health and Science University, Portland, OR USA; 2 OHSU Center for Spatial Systems Biomedicine, Department of Biomedical Engineering, Oregon Health and Science University, Portland, OR USA; 3 Department of Pathology, Oregon Health and Science University, Portland, OR USA; 4 Knight Diagnostic Laboratories, Oregon Health and Science University, Portland, OR USA; 5 Knight Cancer Institute, Oregon Health and Science University, Portland, OR USA; 6 Brenden-Colson Center for Pancreatic Care, Oregon Health and Science University, Portland, OR USA

**Keywords:** Cancer imaging, Image processing, Machine learning

## Abstract

Spatially-resolved molecular profiling by immunostaining tissue sections is a key feature in cancer diagnosis, subtyping, and treatment, where it complements routine histopathological evaluation by clarifying tumor phenotypes. In this work, we present a deep learning-based method called speedy histological-to-immunofluorescent translation (SHIFT) which takes histologic images of hematoxylin and eosin (H&E)-stained tissue as input, then in near-real time returns inferred virtual immunofluorescence (IF) images that estimate the underlying distribution of the tumor cell marker pan-cytokeratin (panCK). To build a dataset suitable for learning this task, we developed a serial staining protocol which allows IF and H&E images from the same tissue to be spatially registered. We show that deep learning-extracted morphological feature representations of histological images can guide representative sample selection, which improved SHIFT generalizability in a small but heterogenous set of human pancreatic cancer samples. With validation in larger cohorts, SHIFT could serve as an efficient preliminary, auxiliary, or substitute for panCK IF by delivering virtual panCK IF images for a fraction of the cost and in a fraction of the time required by traditional IF.

## Introduction

Physicians depend on histopathology—the visualization and pathological interpretation of tissue biopsies—to diagnose cancer. Hematoxylin and eosin (H&E)-stained histologic sections (~ 3–5 μm-thick formalin-fixed paraffin-embedded tissue biopsies) are the standard of care routinely employed by pathologists to make diagnoses. On the basis of the visual attributes that make H&E-stained sections useful to pathologists, a broad field of histopathological image analysis has flourished^1^. Image features derived from H&E-stained sections have been used for tasks ranging from the segmentation of glands in the prostate^2^, grading of breast cancer pathology^3^, to automated classification of early pancreatic cancer^4^. In spite of the rich information highlighted by the non-specific H&E stain, in challenging cases with indeterminate histology or tumor differentiation, antibody labeling of tumor cells by a molecular imaging technique like immunofluorescence (IF) provides further characterization. It is becoming increasingly apparent that determining the spatially-resolved molecular profile of a cancer is important for disease subtyping and choosing a patient’s course of treatment^5^. Despite its clinical value, IF is time- and resource-intensive and requires expensive reagents and hardware, so assessment is typically limited to a small representative section of a tumor, which may not be fully representative of the neoplasm, which can be the case in areas of squamous differentiation in an adenocarcinoma^6^. Also, the cost associated with IF may in some cases limit its use to within highly-developed clinical laboratories, further widening the quality-of-care gap between high- and low-income communities. The gaps between H&E and IF technologies highlight the broader need for automated tools that leverage information attained by a low-cost technology to infer information typically attained by a high-cost technology.

Recent advances in digital pathology and deep learning (DL) have made it possible to automatically extract valuable, human-imperceptible information from H&E-stained histology images^7–11^. In^12^, H&E images and spatially-registered SOX10 immunohistochemistry (IHC) images from the same tissue section were used to train a DL model to infer SOX10 nuclear staining from H&E images alone. Apart from histology and IHC images, other studies have described supervised DL-based methods for inferring fluorescence images from transmitted light images of unlabeled human or rat cell lines or cell cultures^13,14^, but not in complex, associated human tissues. Their methods were also based on supervised pixel-wise learning frameworks, which are known to produce incoherent or discontinuous patterns in the virtual stains for some markers, though some of the authors suggest that an adversarial learning framework could address the problem^13^. We previously introduced an adversarial DL-based method called speedy histological-to-IF translation (SHIFT) and demonstrated its ability to infer fluorescence images from images of adjacent H&E-stained tissue from a single patient with pancreatic ductal adenocarcinoma (PDAC)^15^. To better define the virtual staining problem in the current study, we have developed a serial staining protocol which enables co-registration of H&E and IF data in the same tissue section. In this setting, we test the generalizability of virtual IF staining by SHIFT through model evaluation on PDAC samples from four patients which were selected by an expert pathologist on the basis of their high inter-sample morphological heterogeneity.

DL models require a large amount of heterogeneous training data to generalize well across the population from which the training data was drawn. Since data limitations are common to many biomedical data domains^11,16–18^, we begin by exploring the possibility that the choice of training samples can be optimized by selecting the few samples that are most representative of the population of samples at our disposal. Some DL-based applications have been proposed for histological image comparison and retrieval^19,20^, but to the best of our knowledge none have been proposed for the express purpose of training set selection in a data-limited biomedical imaging domain. We describe the use of a data-driven DL-based method to select samples that optimizes the morphological heterogeneity of the dataset and promotes SHIFT model generalizability.

As a proof of concept, we objectively measure the ability of SHIFT models to infer the spatial distribution of a pan-cytokeratin (panCK) antibody which labels cancer cells. We also show preliminary results for inference on the stromal marker α-smooth muscle actin (α-SMA). By leveraging a morphological signature of a molecular tumor phenotype and proposing feature-guided sample selection for model generalizability, our approach is a small step toward the development of a generalized platform for multiplexed virtual IF imaging of markers in human tissues for which there exists an association between tissue morphology and an underlying molecular phenotype.

## Results

### Building a dataset of spatially-registered H&E and IF images

**Figure 1:**
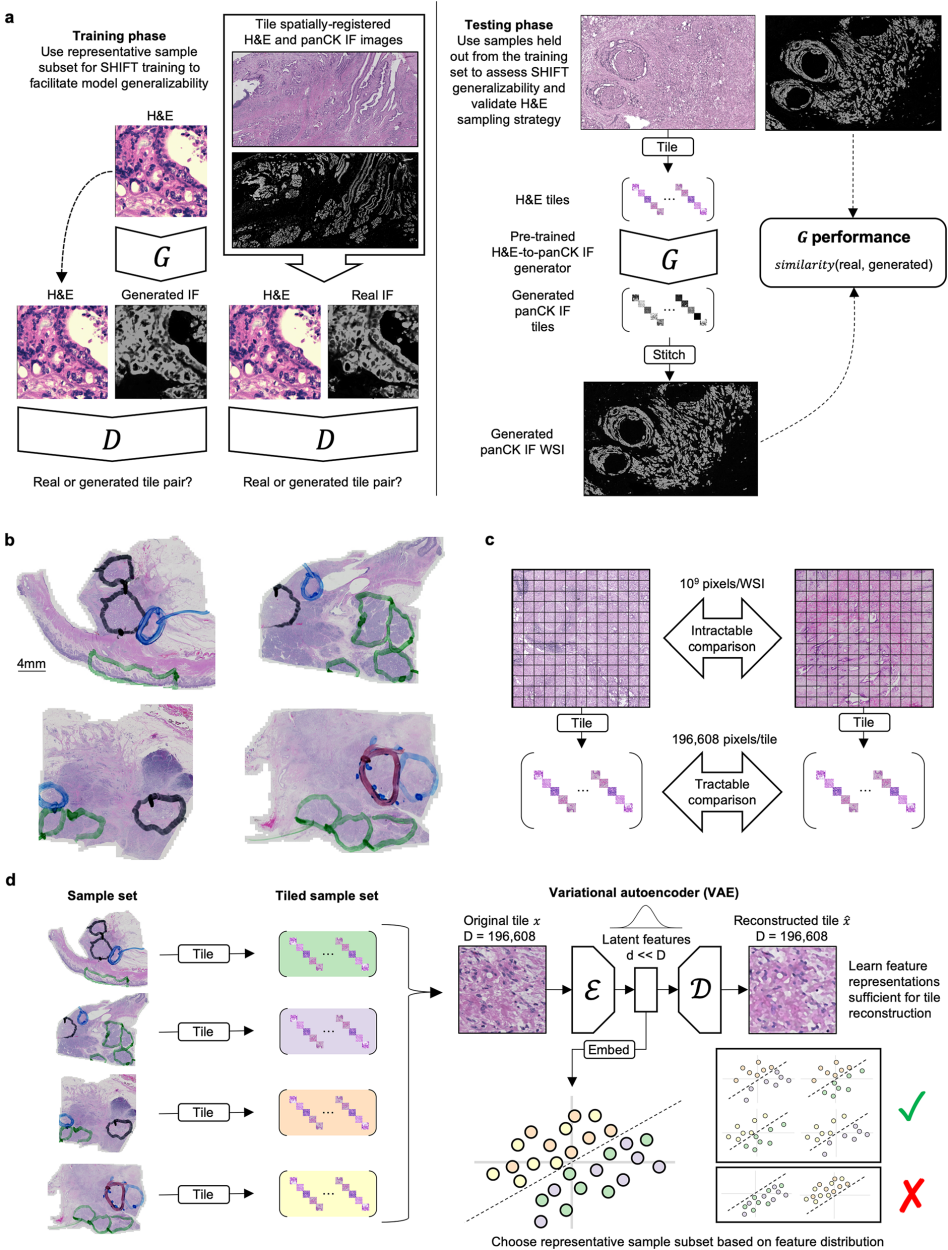
Overview of virtual IF staining with SHIFT and feature-guided H&E sample selection. (**a**) Schematic of SHIFT modeling for training and testing phases. The generator network $G$ generates virtual IF tiles conditioned on H&E tiles. The discriminator network $D$ learns to discriminate between real and generated image pairs. See also Supplementary Fig. S2. (**b**) Four heterogeneous samples of H&E-stained PDAC biopsy tissue used in the current study. Pathologist annotations indicate regions that are benign (green), grade 1 PDAC (black), grade 2/3 PDAC (blue), and grade 2/3 adenosquamous (red). (**c**) Making direct comparisons between H&E whole slide images (WSIs) is intractable because each WSI can contain billions of pixels. By decomposing WSIs into sets of non-overlapping 256 × 256 pixel tiles, we can make tractable comparisons between the feature-wise distribution of tile sets. (**d**) Schematic of feature-guided H&E sample selection. First, H&E samples are decomposed into 256 × 256 pixel tiles. Second, all H&E tiles are used to train a variational autoencoder (VAE) to learn feature representations for all tiles; for each 196,608-pixel H&E tile in the dataset, the encoder ${\mathcal{E}}$ learns a compact but expressive feature representation that maximizes the ability of the decoder ${\mathcal{D}}$ to reconstruct the original tile from its feature representation (see “Methods”). Third, the tile feature representations are used to determine which samples are most representative of the whole dataset.

SHIFT requires spatially-registered pairs of H&E and IF whole slide images (WSIs) for model training and testing (Fig. 1A). Such data would usually be acquired by processing two adjacent tissue sections, one stained by H&E and another stained by IF, then spatially registering the images into the same coordinate system based on their shared features^21^. Unfortunately, this can lead to inconsistencies between H&E and IF image contents when high-frequency cellular features differ between adjacent sections, even when the sections are as few as 5 μm apart. To alleviate this issue, we developed a protocol that allows for H&E and IF staining in the same section of tissue (see “Methods”). Clinical samples of PDAC from four patients (Samples A, B, C and D) were chosen via pathological review of archival H&E images as exemplifying a spectrum of both histological differentiation and heterogeneity (Fig. 1B). Chosen samples were sectioned, processed, and stained with DAPI nuclear stain and panCK monoclonal antibody; the staining was confirmed and the slides were scanned. After scanning, the coverslips were removed and the slides were stained with the designed modified H&E protocol, permanently cover slipped and then scanned again. Nuclear information from the hematoxylin and DAPI stains in pairs of H&E and IF images were used to register images in a common coordinate system. Images were then pre-processed to minimize noise and account for technical variability in staining and image acquisition. To exclude regions of autofluorescence that greatly diminished the signal-to-noise ratio of the real IF images, images from samples B and D were subdivided into image subsets {B1, B2, B3} and {D1, D2, D3, D4, D5}.

### Feature-guided identification of representative histological samples

For a SHIFT model to generalize well across the population of PDAC samples, it must be trained on a representative subset of the population, which motivated the development of a means to quantitatively compare images (see “Methods”). In particular, we wished to learn which sample—or sequence of samples—should be selected to build a training set that is most representative of the population of samples. As a consequence of their large dimensions, direct comparison between gigapixel H&E images is intractable, so we decomposed each image into sets of non-overlapping 256 × 256 pixel tiles (Fig. 1C). Even the small 256 × 256 pixel H&E tiles contain 196,608 (256 × 256 × 3 channels = 196,608) pixel values each and are difficult to compare directly. To establish a more compact but still expressive representation of the H&E tiles, we trained a variational autoencoder (VAE)^22^—an unsupervised DL-based method for representation learning and feature extraction—to learn 16-dimensional feature representations of each tile (see “Methods” for VAE optimization and parameter selection details), which makes comparing tiles more tractable (Fig. 1D).

**Figure 2:**
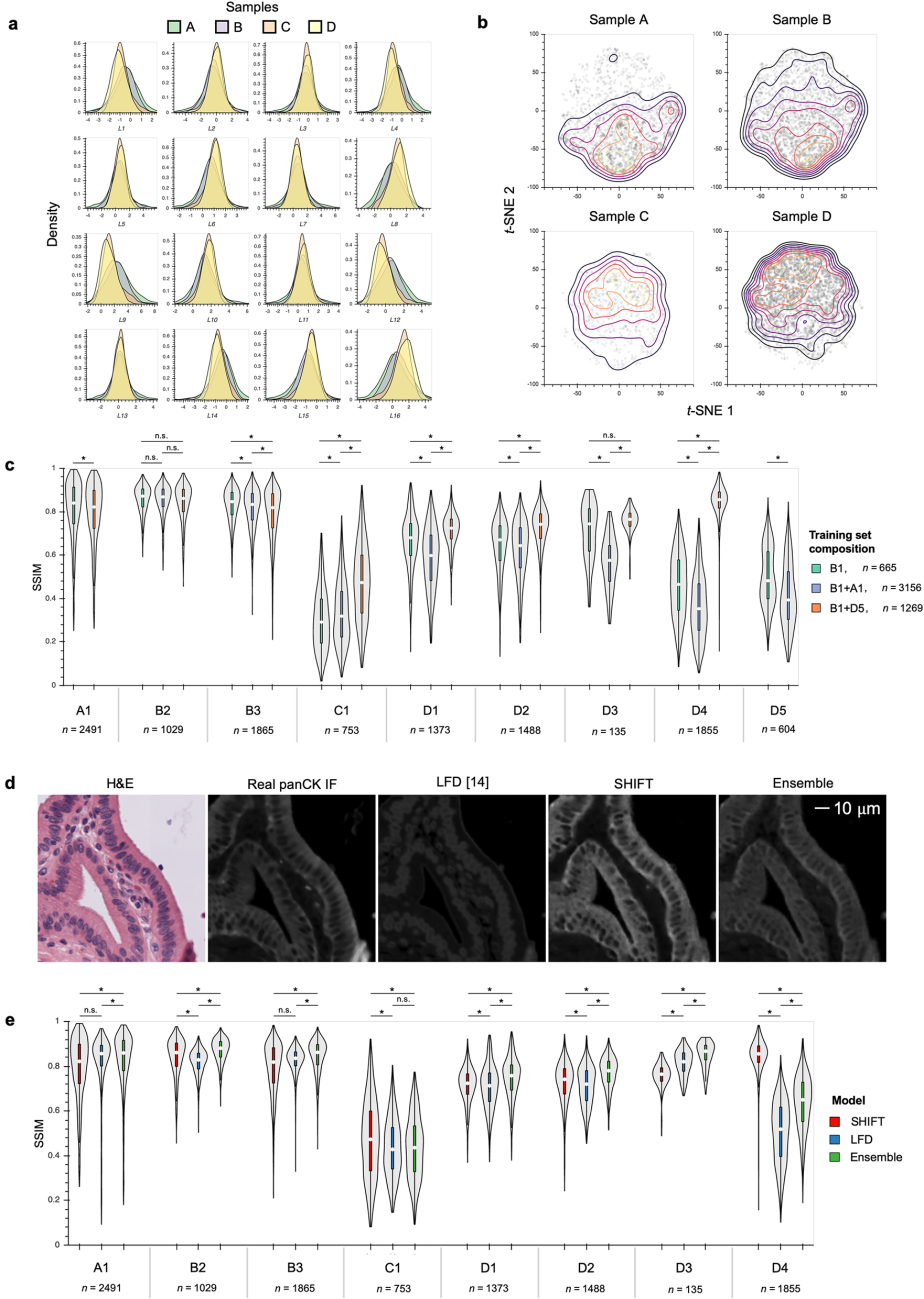
Feature-guided H&E sample selection and virtual IF staining with SHIFT. (**a**) Distribution of the 16 latent features (L1-L16) extracted by VAE from sample H&E tiles. (**b**) *t*-SNE embedding of latent feature representations of sample H&E tiles, faceted by sample identity. Each point in each plot represents a single H&E tile. Contour lines indicate point density. (**c**) SHIFT model test performance for optimal (B and D) and non-optimal (A and B) training set sample compositions. The paired H&E and IF images from samples B and D were subdivided into smaller images B = {B1,B2} and D = {D1,D2,D3,D4,D5} to avoid regions of IF that exhibited substantial autofluorescence. The x-axis labels indicate sample identity, where each letter corresponds to a unique sample and each number corresponds to a subset of that sample. Each *n* denotes the number of image tiles that were extracted from that sample. Plots for sample subsets are not show if that sample subset was a component of a model’s training set. *p < .05; for three group comparisons we used the Friedman test with Nemenyi post-hoc test; for two group comparisons we used the Wilcoxon signed-rank test. White dots in violin plots represent distributional medians. (**d**) Visual comparison of virtual staining methods. The ensemble results are attained by averaging the output images of SHIFT and Label-Free Determination (LFD) models. See also Supplementary Fig. S2. (**e**) Test performance comparison of virtual staining methods. The x-axis labels indicate sample identity, where each letter corresponds to a unique sample and each number corresponds to a subset of that sample. Each *n* denotes the number of image tiles that were extracted from that sample. Plots for sample subsets B1 and D5 are not show because those sample subsets were components of the models’ training sets. *p < .05; Friedman test with Nemenyi post-hoc test. White dots in violin plots represent distributional medians.

Using the VAE which we pre-trained on all samples, we extracted features from each H&E tile and assessed how each feature was distributed across samples, finding that several of the features discriminated between samples (Fig. 2A). In particular, the bimodal distribution of some features suggested two sample clusters, one formed by samples A and B, and another formed by samples C and D. These clusters were corroborated by visualization of the sample tiles embedded in the reduced-dimension feature space generated by *t*-SNE^23^ (Figs. 1D and 2B). The *t*-SNE embedding is strictly used as a visual aid and validation of the quantitative selection of the most representative set of samples by the algorithm based on the full 16-dimensional VAE features. Since the feature distributions of the H&E tiles highlighted the redundancy between clustered samples, we reasoned that a balanced selection of samples from each cluster would yield a more representative training set and ultimately improve SHIFT model generalizability. Using the full 16-dimensional feature representations of the H&E tiles and an information-theoretic framework for representative sample selection^24^ (see “Methods”), we were able to quantitatively identify sample B and the duo of samples B and D as the single and two most representative samples, respectively, which were then considered for training sets in subsequent experiments. Supplementary Fig. S1 illustrates the feature distributions of several sample combinations in comparison to that of the full dataset.

### Virtual IF staining in histological samples

SHIFT models are built on an adversarial image-to-image translation framework^25^, with a regularization strategy designed to improve inference on sparse IF images (Fig. 1A and Supplementary Fig. S2; see “Methods”). Adversarial learning frameworks compute their losses over images, in contrast to strictly supervised learning frameworks where losses are computed over pixels, which has been suggested as a means to improve model inference on virtual staining tasks^13^. Having identified the most representative samples in our dataset, we next tested whether or not a SHIFT model could learn a correspondence between H&E and IF images that generalizes across samples.

We hypothesized that if tissue and cell morphologies observed in H&E-stained tissue are a function of a given marker, then it should be possible to infer the spatial distribution of that marker based on the H&E-stained tissue alone; that is, H&E-to-IF translation should be learnable and generalizable such that a model can be extended to samples from patients that were not included in the training set. To test this hypothesis, we trained SHIFT models to generate virtual IF images of the cancer marker panCK conditioned on input H&E images alone (Fig. 1A). To simultaneously assess the utility of our sample selection method, we trained models using different combinations of sample subsets in the training set. Training sets consisted of paired H&E and IF image tiles from either sample subset B1 (from the most representative single sample), sample subsets B1 and D5 (from the most representative duo of samples), or sample subsets A1 and B1 (from a less representative duo of samples as counterexample). Sample subsets B1 and D5 were selected because they contained a similar number of tiles, providing a balance between the sample clusters. Once trained, SHIFT models are capable of translating H&E WSIs into virtual IF WSIs in tens of seconds (see “Methods”). Model performance was quantified by measuring the structural similarity (SSIM)^10,26^, a widely used measure of image similarity as perceived by the human visual system, between corresponding virtual and real IF images from samples left out of the model’s training set. The SSIM between two images is calculated over pixel neighborhoods in the images and provides a more coherent measure of image similarity than pixel-wise measures like Pearson’s *r*. Considering the SSIM performance of the model trained on B1 as the baseline, we see a significant improvement in model generalizability on held-out sample C (Friedman statistic = 428.4, *p* = 9.6E−94) and held-out subsets D1 (Friedman statistic = 587.4, *p* = 2.7E−128), D2 (Friedman statistic = 298.0, *p* = 2.0E−65), and D4 (Friedman statistic = 3099.1, *p* < 2.2E−308) when the more representative sample subsets B1 and D5 are used for training than when the less representative sample subsets A1 and B1 are used (Fig. 2C). By stitching together the virtual IF tiles from a given sample in the test set, we were able to make large-scale comparisons between real and virtual panCK IF images (Supplementary Fig. S3). We also experimented with SHIFT inference of the stromal marker α-SMA (Supplementary Fig. S4).

SHIFT is not the only virtual staining method to have been recently proposed. Label-free determination (LFD)^14^ is a supervised DL-based virtual staining method which produces models that were shown to have learned the relationship between images of cell cultures visualized by transmitted light or fluorescence, where sub-cellular structures have been labeled with genetically-encoded fluorescent tags. Because the SHIFT generator *G* and the LFD are both based on the popular U-net architecture^27^, we compared these models that generate images using a similar architecture, but have differing training formulae and loss functions (see “Methods”). To make a fair comparison between the adversarial SHIFT and supervised LFD models, we trained a LFD using the representative sample subsets B1 and D5, matching the number of optimization steps taken by the SHIFT model that was trained using the same training set (Supplementary Fig. S5). In addition to the performance of independent SHIFT and LFD models, we also considered the ensemble result, taken as the average image of the SHIFT and LFD output images (Fig. 2D). Across all samples in the test set, either SHIFT alone or the ensemble of SHIFT and LFD tended to perform better than LFD alone (Fig. 2E). In addition to comparing SHIFT and LFD models, we also tried removing the discriminator and adversarial loss term from the panCK SHIFT model, leaving just the U-net generator. We find that these models trained using the pixel-wise L1 loss alone produced virtual panCK staining with good localization, but poor resolution of finer cellular structure, highlighting the importance of the adversarial loss for producing realistic virtual stains (Supplementary Fig. S6).

## Discussion

Spatially-resolved molecular profiling of cancer tissues by technologies like IF provides more information than routine H&E histology alone. However, the rich information obtained from IF comes at significant expense in time and resources, restricting IF access and use. Here, we present and extend the validation of SHIFT, a DL-based method which takes standard H&E-stained histology images as input and returns virtual panCK IF images of inferred marker distributions. Using a limited but heterogeneous dataset, we demonstrated that SHIFT models are able to generalize across samples drawn from different PDAC patients, even for training sets that are over an order of magnitude smaller than the test set (train $$n = 665 $$ and test $$n = 11,593$$ for models trained on sample subset B1 only). Results from our sampling experiments are consistent with the expectation that an automated and quantitative method for representative sample selection will be critical to the effective development and deployment of DL models on large-scale digital pathology datasets. Finally, we compared the adversarial SHIFT method with an alternative, supervised virtual staining method and found that the virtual staining task tends to be best accomplished by the ensemble of both methods. With the incorporation of an adversarial loss term, a SHIFT model computes loss over images, rather than strictly over pixels as do other virtual staining methods^13,14^, which may explain its positive contribution to the model ensemble. Based on the success of DL-based ensemble methods in other biomedical domains^18,28^, we expect ensemble methods to become increasingly relevant to the development of virtual staining applications.

While we have demonstrated the application of SHIFT for the estimation of an IF tumor signature, there are emerging opportunities to test the extensibility of our approach and relate it to the approaches of others. In particular, a comparison between SHIFT and methods which estimate single-stain IHC status conditioned on H&E images^12,29^ would be insightful, since it remains unclear whether there is value added in learning from either IF or chromogenic signals. Following recent advances in multiplexed immunofluorescence and immunohistochemistry (mIF/IHC) technology^30–33^, it is now possible to visualize tens or hundreds of distinct markers in a single tissue section. On their own, these technologies promise a more personalized medicine through a more granular definition of disease subtypes, and will undoubtedly broaden our understanding of cellular heterogeneity and interaction within the tumor microenvironment, both of which play increasingly important roles in the development and selection of effective treatments^34,35^. With a paired H&E and mIF/IHC dataset that encompasses the expression of hundreds of markers within the same (or serially-sectioned) tissue, we could begin to quantify the mutual information between histology and expression of any marker of interest. Notwithstanding the prospect of virtual multiplexing, virtual panCK IF alone could be of useful to spatial profiling platforms which use panCK IF to label tumor regions for localized spatial profiling of protein and RNA abundances in formalin-fixed tissues^36^.

There are obvious limitations and challenges to both the feature-guided sampling and virtual staining methods we present here. Both methods assume an association between H&E and IF representations of tissue. Since this is unlikely to be the case in general, determining which markers have a histological signature will be essential to the evaluation of clinical utility for virtual staining methods. For markers without a H&E-to-IF association, our methods may fail to maximize representativeness or make incorrect estimates of IF signals. This should be seen as a feature rather than failure of our methods, since they provide a means of quantitatively delineating markers that have a H&E-to-IF association from those that do not. Notwithstanding, even when there is an association, finding a meaningful way to compare real and virtual images remains a challenge, as we have experienced in experiments modeling markers with fine, high-frequency distributions like α-SMA (Supplementary Fig. S4). Like other virtual staining methods that have been deployed on whole human tissues^9,10^, we used SSIM as a measure of image similarity between real and virtual IF images. This classical perceptual measure is used in many imaging domains, but we found that it is sensitive to perturbations commonly associated with image registration and technical or instrumentation noise (Supplementary Fig. S7). In light of this, we advocate for the development and use of perceptual measures that are more aware of such perturbations and better correlate with human perception of image similarity or quality^37,38^.

It must be restated that our results are supported by a dataset comprised of samples from just four patients, so some fluctuation in performance between samples should be expected, and indeed was observed (Supplementary Fig. S3). Our goal in choosing a relatively small dataset was to demonstrate that, even when limited, SHIFT could learn a general relationship between H&E and IF tissue representations, and we believe that the fluctuation in performance between samples could be addressed by increasing the sample size. With the emergence of digital pathology datasets containing tens of thousands of whole slide images^11^, the opportunities to improve virtual staining technologies are only becoming more numerous.

In spite of representing four patients, the samples in our study were selected by a board-certified pathologist to be as heterogenous as possible, encompassing the spectrum of PDAC morphology, albeit in as few samples as possible. While we used these heterogeneous and associated human tissues for our study, other virtual staining methods have only been demonstrated on relatively homogeneous human or rat cell lines^13,14^ and with far less total image area (Supplementary Table S1). Given the precedent set by these prior works, we feel that the work we present here is within scope as a proof-of-concept study of sample selection and histology-based IF prediction in a digital pathology application. Moreover, the protocol that we developed to allow H&E and IF staining in the same tissue sections will be of significant value to the community, since spatially-paired H&E and IF data is difficult to generate from adjacent sections due to tissue deformation and cellular discontinuity between sections.

Since SHIFT can infer virtual panCK IF images as H&E-stained tissue section are imaged, SHIFT could provide pathologists with near-real-time interpretations based on standard H&E-stained tissue in augmented settings. Therefore, SHIFT could serve as an efficient preliminary, auxiliary, or substitute technology for traditional panCK IF in both research and clinical settings by delivering comparable virtual panCK IF images for a fraction of the cost and in a fraction of the time required by traditional IF or mIF/IHC imaging. With clinical validation in larger cohorts, the advantages of a SHIFT model over traditional IF would include (1) eliminating the need for expensive imaging hardware, bulk reagents, and technical undertaking of IF protocols; (2) virtual IF images can be generated in near-real time; and (3) the portability of SHIFT allows it to be integrated into existing imaging workflows with minimal effort. As such, we see further validation of SHIFT as an opportunity to simultaneously economize and democratize advanced imaging technologies in histopathology workflows, with implications for multiplexed virtual imaging. Further, we see our methods for the optimal selection of representative histological images, which promote morphological heterogeneity in the training dataset as well as reduce unnecessary effort on IF staining, as a complement to data augmentation, transfer learning, and other means of addressing the problem of limited training data. This will contribute to saving resources and minimizing unnecessary efforts to acquire additional staining or manual annotation for DL applications in biomedical imaging.

## Methods

Resources table.

**Table:** 

Reagent or resource	Source	Identifier
**Antibodies**		
Alpha-smooth muscle actin monoclonal antibody (1A4 (ASM-1))	ThermoFisher Scientific	https://www.thermofisher.com/antibody/product/Alpha-Smooth-Muscle-Actin-Antibody-clone-1A4-asm-1-Monoclonal/MA5-11547
Goat anti-mouse IgG2a cross-adsorbed secondary antibody, AlexaFluor 555	ThermoFisher Scientific	https://www.thermofisher.com/antibody/product/Goat-anti-Mouse-IgG2a-Cross-Adsorbed-Secondary-Antibody-Polyclonal/A-21137
Pan-cytokeratin monoclonal antibody (AE1/AE3), AlexaFluor 488, EBioscience	ThermoFisher Scientific	https://www.thermofisher.com/antibody/product/Pan-Cytokeratin-Antibody-clone-AE1-AE3-Monoclonal/53-9003-82
Ki-67 (D3B5) monoclonal antibody, AlexaFluor 647, Conjugate #12075	Cell Signaling Technology	https://www.cellsignal.com/products/antibody-conjugates/ki-67-d3b5-rabbit-mab-alexa-fluor-647-conjugate/12075?N=4294960176&Nrpp=100&fromPage=plp
**Biological samples**		
Human PDAC tumor samples	Oregon Pancreas Tissue Registry	IRB: 3609
**Chemicals, peptides, and recombinant proteins**		
SlowFade Gold Antifade Mountant with DAPI	ThermoFisher Scientific	https://www.thermofisher.com/order/catalog/product/S36938
Normal goat serum blocking solution	Vector laboratories	https://vectorlabs.com/normal-goat-serum-blocking-solution.html
**Deposited data**		
Imaging data	This manuscript	https://gitlab.com/eburling/shift/
**Software and algorithms**		
Python	Python Software Foundation	RRID:SCR_008394
PyTorch	Paszke et al.^39^	https://pytorch.org/
imgaug	Jung^40^	https://github.com/aleju/imgaug
Netron	https://www.lutzroeder.com/ai/	https://github.com/lutzroeder/netron
HoloViews	Stevens et al.^41^	https://holoviews.org/
Bokeh	Bokeh Development Team^42^	https://bokeh.pydata.org/
scikit-image	van der Walt et al.^43^	https://scikit-image.org/
scikit-learn	Pedregosa et al.^44^	https://scikit-learn.org
scikit-posthocs	Terpilowski et al.^45^	https://doi.org/10.21105/joss.01169
SciPy	Virtanen et al.^46^	https://www.scipy.org/
Jupyter	Kluyver et al.^47^	https://jupyter.org/

### Materials availability

Further information and requests for resources should be directed to and will be fulfilled by the corresponding author, Dr. Young Hwan Chang (chanyo@ohsu.edu).

### Experimental model and subject details

#### Human tissue samples

Four cases of moderately differentiated pancreatic ductal adenocarcinoma (PDAC) were retrieved from the Oregon Health & Science University (OHSU) Surgical Pathology Department under the Oregon Pancreas Tissue Registry (IRB00003609). Informed written consent was obtained from all subjects. All experimental protocols were approved by the OHSU Institutional Review Board. All methods were carried out in accordance with relevant guidelines and regulations. Sample A was from a male aged 83 at diagnosis; sample B was from a female aged 74 at diagnosis; sample C was from a female aged 57 at diagnosis; and sample D was from a female aged 73 at diagnosis. H&E-stained sections were secondarily reviewed by two board-certified surgical pathologists tasked to identify and classify areas of tumor heterogeneity in representative sections from each case. Discrepancies between pathologists were ameliorated by consensus review. Samples were chosen via pathological review as exemplifying a spectrum of both histological differentiation and heterogeneity.

### Clinical method details

#### Pathological evaluation of human tissue samples

Gold standard review of histologic sections by pathologists tasked with identifying heterogeneous differences in PDAC tumor morphology and grade revealed interobserver agreement in the identification of areas of squamous differentiation in one case and various tumor grades within neoplasms in the other three cases. All four cases were predominantly grade 2 adenocarcinoma and there was no disagreement evaluating marked regions of interest. The case with areas of squamous differentiation did not clearly meet the 30% threshold for adenosquamous classification. The other three cases were predominantly grade 2 with foci of grade 1 and others with grade 3.

### Immunofluorescence staining and image processing

#### Preparation of tissue for immunofluorescence staining

Formalin-fixed paraffin-embedded tissue blocks were serially sectioned by the OHSU Histopathology Shared Resource. From each block, three sections were cut in order to generate a standard H&E for pathological review and downstream analysis, a second serial section of tissue for immunofluorescence staining/post-immunofluorescence H&E staining, and a third section for secondary only control. After sectioning, the second serial tissue section was immediately baked at 55 °C for 12 h and subjected to standard deparaffinization; the slides underwent standard antigen retrieval processing, washing, and blocking. Upon completion, primary antibodies were diluted and applied.

#### Application of antibodies

Alpha-Smooth Muscle Actin (Mouse monoclonal antibody, IgG2a, Clone: 1A4; Pierce/Invitrogen, cat#MA5-11547) was diluted to 1:200 with Ki-67 (D3B5), (Rabbit monoclonal antibody, IgG, Alexa Fluor 647 Conjugate; Cell Signaling Technology, cat#12075S) diluted to 1:400, along with Pan Cytokeratin (AE1/AE3) (Mouse monoclonal antibody, IgG1, Alexa Fluor 488 Conjugate; ThermoFisher, cat#53-9003-82), which was diluted to 1:200 in 10% Normal Goat Serum in 1% Bovine Serum Albumin in Phosphate Buffered Saline. Primary antibodies were diluted and incubated overnight at 4 °C. After incubation, secondary antibody (Goat anti-mouse monoclonal antibody, IgG2A, Alexa Fluor 555 Conjugate; Life Technologies, cat# A21137), at 1:200 dilution was applied to the slides and incubated at room temperature for one hour. After incubation slides were washed and mounted with Slowfade Gold Antifade Mountant with DAPI (Fisher Scientific, cat#S36936) in preparation for image acquisition.

#### Post-IF H&E staining of tissue samples

After the IF stained slides were scanned and the immunofluorescence staining verified, the glass coverslips were removed and the slides were immediately processed for post-IF H&E staining. Post-IF H&E staining was performed with the Leica Autostainer XL staining system at the OHSU Histopathology Shared Resource with the modified staining protocol described in the table below:

**Table:** 

Hematoxylin	10 min
Wash in water	1 min
Acid alcohol (0.5% HCl in 70% Ethanol)	8 s
Wash in water	25 s
Bluing solution	2 min
Wash in water	20 s
80% Ethanol/water	25 s
Eosin	10 s
80% Ethanol/water	25 s
95% Ethanol/water	20 s
100% Ethanol (two times)	25 s
Xylene (five times)	25 s

#### Image acquisition and presentation

Slides were scanned with the Zeiss Axio Scan.Z1 slide scanner of the OHSU Advanced Multiscale Microscopy Shared Resource with the 20X objective in both brightfield and immunofluorescence scanning. Carl Zeiss Images (CZI) were acquired using Zeiss Zen software. CZI images from the Zeiss Axioscan Slide Scanner were processed with the Zeiss Blue Zen Lite microscope software package. All brightfield and immunofluorescence images were manually annotated and exported as TIFF files for downstream image processing.

#### Image pre-processing

Raw H&E and IF whole slide images (WSIs) must be pre-processed to remove technical noise, account for between-sample intensity variation, and align paired H&E and IF WSIs in a shared coordinate system. To do so, we use the following pipeline:

1. Quality control: formalin-fixed pancreatic tissue is prone to high levels of autofluorescence, which can mask specific IF signal. Regions of WSIs which exhibited low IF signal-to-noise due to autofluorescence as determined by pathologist review were excluded from our analysis. Divisions of samples B and D were based on the geometries of the image regions determined unaffected by autofluorescence. Some acceptable regions like D3, which contained 90 tiles, were relatively small due to surrounding regions of autofluorescence.
2. Downscaling: 20X WSIs are downscaled by a factor of 2 in x and y dimensions to generate 10X WSIs. We experimented with using either 20X or 10X images and found that models performed best when using 10X images.
3. Registration: H&E and IF WSIs are spatially registered using an affine transformation that is estimated using matched SURF features^21,48^ extracted from hematoxylin and DAPI binary masks of nuclei generated by Otsu’s thresholding method, respectively. Concretely, registration of an H&E WSI and a corresponding IF WSI of the same tissue was achieved using MATLAB^49^ through the following steps:
  i. Conversion of H&E images from RGB colorspace to grayscale using the MATLAB function rgb2gray.
  ii. Binarization and complementation of the grayscale H&E and DAPI WSIs using the MATLAB functions imbinarize and imcomplement, creating nuclei masks from each of the H&E and DAPI WSIs.
  iii. Detection and extraction of SURF features from each of the H&E and DAPI nuclei masks using the MATLAB functions detectSURFFeatures, selectStrongest, and extractFeatures. We constrained the number of features selected to min(10,000, number of features detected) to reduce the computational cost of feature matching in the next step.
  iv. Feature matching between the features extracted from the H&E and DAPI nuclei masks using the MATLAB function matchFeatures.
  v. Estimation and application of the affine transformation matrix which correctly registers H&E and DAPI nuclei masks using the MATLAB functions estimateGeometricTransform and imwarp. The same transformation which correctly registers DAPI to the H&E WSI is used to register IF WSIs.
4. Technical noise reduction: IF WSIs are median filtered with a 5-pixel-radius disk structuring element.
5. Intensity normalization: H&E WSI pixel intensities are normalized as previously described^50^. Following Christiansen et al.^13^, IF WSI pixel intensities are normalized to have a fixed mean = 0.25 and standard deviation = 0.125, then clipped to fall within (0,1).
6. Image tiling: WSIs are tiled into non-overlapping 256 × 256 pixel tiles, such that each H&E tile has a corresponding spatially-registered IF tile. H&E tiles that contained more than 50% background pixels were removed along with the corresponding IF tiles. Background pixels were defined as those with 8-bit RGB intensities all greater than 180. Each 10X WSI is comprised of hundreds or thousands of non-overlapping 256 × 256 pixel tiles.

### Feature-guided training set selection

Although DL approaches like SHIFT and Label-Free Determination require substantial training data to be robust and generalizable, due to resource constraints we hope that a small number of paired H&E and IF image samples is required for model training. Typically, archival WSIs of H&E-stained tissue sections exist on-hand for each sample, which allows for the screening of samples to identify the minimal number of samples that maximally represent the morphological spectrum of the disease being considered. Recent studies demonstrate that DL systems are well-suited for image retrieval tasks in digital pathology^19,20^, wherein a pathologist submits a query image or region of interest and the DL system returns similar images based on their DL-defined feature representations. We seek to solve the inverse task of heterogeneous training set selection in digital pathology, though our approach could be extended to any data-limited biomedical imaging domain.

Since PDAC is a morphologically heterogeneous disease, building a representative training set is crucial to the design of a model that will generalize across heterogeneous biopsy samples after deployment. In order to minimize the required resources for acquiring paired H&E and IF images but still cover a broad spectrum of heterogeneous morphological features in the selected H&E samples, we propose a clustering method to learn a heterogeneous representation of H&E sample images. To assess the morphological features of each sample, we use a variational autoencoder (VAE)^22^ to extract 16-dimensional feature vectors from each H&E tile to establish comparisons between samples. Since texture and morphological features on H&E tiles in each cluster of samples will be comparatively more similar than those of the other cluster, we only select representative H&E samples from each cluster for our training dataset. We also tried using other feature vector sizes for representation learning, e.g. 2, 4, 8, 32, but found that a feature vector size of 16 yielded the lowest reconstruction losses.

For example, if there are four samples being considered for IF staining, but resources limit the number of samples that can be stained to two, a decision must be made about which samples should be selected. For the four samples, we aggregate their archival H&E WSIs, extract features from H&E tiles for each sample using a VAE, and quantitatively determine the samples needed to maximally cover the feature space over which the H&E tile set is distributed. By screening and selecting samples in this data-driven fashion, we exclude homogeneous or redundant samples that would not contribute to model generalizability. This maximizes model performance by ensuring that our training dataset is representative of the disease being modeled, thus minimizing cost through the selection of the fewest samples required to do so. Perhaps more importantly, when we fail to generate reliable virtual IF images for certain tissue samples or IF markers, this framework will be useful to examine whether or not their morphological features are well presented in the training dataset, which can guide how we select additional samples when updating our dataset.

To identify the sequence of samples that should be selected, we adapt an information-theoretic sample selection algorithm^24^ which is more capable of generating representative subsets of data with imbalanced features than other classical algorithms used for sample selection, like maximum coverage^51^ or k-medoid clustering^52^. The algorithm is parameterized using the following notation:

**Table:** 

Parameter	Description
$X$	Complete tile set of all samples,$X = \left\{ {x_{1} , x_{2} , \ldots , x_{n} } \right\}$
$x_{i}$	Single tile,$x_{i} \in X$
$X_{i}$	Subset of $X$ corresponding to the $i$th sample,$X_{i} \subset X$
$F$	Complete VAE-learned feature set,$F = \left\{ {f_{1} , f_{2} , \ldots , f_{m} } \right\}$
$f_{i}$	Single feature,$f_{i} \in F$
$A$	Random variable defined over $F$
$T$	Random variable defined over $X$

We begin with a tiles ✕ features table, where we set $m = 16$ for our experiments:

**Table:** 

	$f_{1}$	$f_{2}$	…	$f_{m}$
$x_{1}$	− 1.64266	1.36952	…	1.23509
$x_{2}$	− 0.792104	− 0.481497	…	1.07938
…	…	…	…	…
$x_{n}$	0.00163981	− 0.0162441	…	-0.95883

We normalize across rows of the table, such that each tile is now represented as a probability distribution over the feature domain:

**Table:** 

	$f_{1}$	$f_{2}$	…	$f_{m}$	Sum
$x_{1}$	0.00418311	0.0850498	…	0.0743519	1
$x_{2}$	0.0148384	0.0202433	…	0.0964193	1
…	…	…	…	…	1
$x_{n}$	0.0208721	0.0205021	…	0.00798802	1

We define the random variables $T$ and $A$ over tile domain $X$ and the feature domain $F$, respectively, such that $P(A = f_{1} |T = x_{1} ) = 0.418311$, $P(A = f_{2} |T = x_{2} ) = 0.0202433$. , and so on. With this conditional probability table, we can define probability distributions each subset $X_{i}$: $P(A|X_{i} ) = \frac{1}{{|X_{i} |}}\mathop \sum \limits_{{x \in X_{i} }}^{ } P(A|x).$ To measure the representativeness of sample $X_{i}$ to the full dataset $X$, we compute the Kullback–Leibler (KL) divergence between $P(A|X_{i} )$ and $P(A|X)$: $KL\left( {P(A|X_{i} )||P(A|X)} \right) = \mathop \sum \limits_{f \in F}^{ } P\left( { f | X_{i} } \right)log\frac{{P\left( { f | X_{i} } \right)}}{P( f | X)}.$ We then weight this divergence by the proportion of $X$ that $X_{i}$ comprises,$\frac{{\left| {X_{i} } \right|}}{\left| X \right|}$, to prioritize subsets that contribute many tiles to $X$. We define the single most representative sample as $\hat{X}_{1} = min_{{X_{i} \subset X}} \left( {\frac{\left| X \right|}{{\left| {X_{i} } \right|}}KL\left( {P(A|X_{i} )||P(A|X)} \right)} \right),$ the most representative duo of samples as $\hat{X}_{2} = \hat{X}_{1} + min_{{X_{i} \subset X - \hat{X}_{1} ,}} \left( {\frac{\left| X \right|}{{\left| {X_{i} } \right|}}KL\left( {P(A|X_{i} + \hat{X}_{1} )||P(A|X)} \right)} \right),$ the most representative trio of samples as $\hat{X}_{3} = \hat{X}_{2} + min_{{X_{i} \subset X - \hat{X}_{2} ,}} \left( {\frac{\left| X \right|}{{\left| {X_{i} } \right|}}KL\left( {P(A|X_{i} + \hat{X}_{2} )||P(A|X)} \right)} \right),$ and so on. In this way, we define the sequence of samples that should be chosen to optimally increase the representativeness of the training set.

### Model architectures

#### Conditional generative adversarial networks

Image-to-image translation—the mapping of pixels from one scene representation to pixels of another representation of the same scene—is a fundamental image processing problem. Conditional generative adversarial networks (cGANs)^53,54^ are a compelling deep learning-based solution to the image-to-image translation problem which have been deployed for many tasks, including detection of skin lesions^16^, retinal image synthesis^17^, super-resolution fluorescence image reconstruction^55^, and virtual H&E staining^10^. To approach the problem of translating H&E images to their IF counterparts, SHIFT adopts the cGAN-driven architecture *pix2pix*^25^, which benefits from its bipartite formulation of generator and discriminator. Like other methods proposed for image-to-image translation, cGANs learn a functional mapping from input images $$x$$ to ground truth target images $$y$$, but, unique to a cGAN architecture, it is the task of a generator network $$G $$ to generate images *ŷ* conditioned on $$x$$, i.e. $$G\left( x \right)$$ = *ŷ*, that fool an adversarial discriminator network $$D$$, which is in turn trained to tell the difference between real and generated images. What ensues from this two-network duel is a $$G$$ that generates realistic images that are difficult to distinguish from real images, some GAN-generated images being sufficiently realistic to be considered as a proxy for the ground truth when labeled data are scarce or prohibitively expensive. Concretely, the cGAN objective is posed as a binary cross-entropy loss:

$$ {\mathcal{L}}_{{{\text{cGAN}}}} \left( {G,D} \right) = {\mathbb{E}}_{{x,y\sim p_{{data\left( {x,y} \right)}} }} \left[ {\log D\left( {x,y} \right)} \right] + {\mathbb{E}}_{{x\sim p_{data\left( x \right)} }} \left[ {\log \left( {1 - D\left( {x,G\left( x \right)} \right)} \right)} \right] $$

where $G$ seeks to minimize the objective and thus minimize the distinguishability of generated and real images, while $D$ seeks the opposite. In addition to the task of fooling $D$, $G$ is also encouraged to generate images that resemble real images through incorporation of an L1 reconstruction loss term:

$$ {\mathcal{L}}_{{{\text{L}}1}} \left( G \right) = {\mathbb{E}}_{{x,y\sim p_{{data\left( {x,y} \right)}} }} \left[ {y - G\left( x \right)_{1} } \right] $$

The full cGAN objective is:

$$ G^{*} = {\arg}\mathop {\min }\limits_{G} \mathop {\max }\limits_{D} {\mathcal{L}}_{{{\text{cGAN}}}} \left( {G,D} \right) + \lambda {\mathcal{L}}_{{{\text{L}}1}} \left( G \right) $$

where the L1 tuning parameter λ = 100 is adapted according to the IF stain prevalence in the current batch of IF tiles^15^ i.e. if 50% of the pixels in the current batch of IF tiles are positively stained above the mean intensity of the WSI, then λ  = 100 × 0.5 = 50. Training data consist of spatially registered pairs of H&E image tiles (*x*) and IF image tiles (*y*), while the test data consist of H&E and IF image pairs withheld from the training data. Models were trained using the Adam optimizer with a learning rate of 0.002 for 500 epochs. Training batch sizes were set to 64. The first layers of both the generator and discriminator networks were 128 filters deep (see Supplementary Fig. S2 for additional architectural details). Models were trained and tested using a single NVIDIA V100 graphics processing unit (GPU). Once trained, models were capable of processing a 10X (0.44 microns/pixel) H&E image tile containing 256 × 256 pixels into its corresponding virtual IF tile in 10 microseconds, corresponding to a virtual staining rate of 22 mm^2^ tissue per second, or approximately one virtual IF WSI generated per 20 s. Full model details are available at https://gitlab.com/eburling/shift/.

*Variational autoencoder*s.

The VAE architecture^22^ is designed to elucidate salient features of data in a data-driven and unsupervised manner. A VAE model seeks to train a pair of complementary networks: an encoder network θ that seeks to model an input *x*_*i*_ as a hidden latent representation *z*_*i*_, and a decoder network ϕ that seeks to reconstitute *x*_*i*_ from its latent representation *z*_*i*_. The VAE cost function shown below penalizes model training with an additional Kullback–Leibler (KL) divergence term that works to conform the distribution of *z* with respect to a given prior, which in our case is the standard normal distribution:

$$ {\mathcal{L}}_{i} \left( {x_{i} ,\theta ,\phi } \right) = - {\mathbb{E}}_{{z\sim q_{\theta } \left( {z|x_{i} } \right)}} \left[ {\log p_{\phi } \left( {x_{i} |z} \right)} \right] + {\text{KL}}\left( {q_{\theta } \left( {z|x_{i} } \right)p\left( z \right)} \right) $$

where

$$ p\left( z \right) = {\mathcal{N}}\left( {0,1} \right) $$

By specifying a latent dimension *z* less than the input dimension of *x*, a VAE model learns a pair of optimal encoding and decoding functions that enable reconstruction of an input sample subject to capacity constraints of the latent feature space within the model. In general, this formulation learns encoding functions that compress the information content in the high-dimensional input into a low-dimensional embedding space that learns dataset features sufficient to reconstitute the original input sample while preserving an expected distribution over the learned features. This interpretation enables a specified selection criteria function designed to sample whole slide images whose constituent tiles maximally cover the entire learned feature space with a minimal number of samples.

#### Model ensembles

In addition to testing the ability of independent SHIFT and LFD models to generate virtual IF images, we also tested model ensembles. Ensemble images were generated by simply averaging the virtual IF image outputs of SHIFT and LFD models trained to generate the same stain using the same training set.

#### Imaging data augmentation

To boost the effective number of images in our training sets and improve model robustness against expected types of technical noise, we apply image augmentations to each image in each training batch using the Python library imgaug^40^. We apply Gaussian blur, flipping, affine geometric transformation, Gaussian noise, Poisson noise, rotation, and add to hue and saturation in each channel. The implementation of our imaging data augmentation can be viewed at https://gitlab.com/eburling/shift.

#### Image processing in figures

The IF images in Fig. 1 are contrast enhanced by saturating the top 1% and bottom 1% of pixel intensities. All other images are processed as described in the image pre-processing section above.

### Quantification and statistical analysis

#### Image comparisons

To compare real and virtual IF images, we measure their structural similarity^26^ using the compare_ssim function implemented in the Python library scikit-learn^44^. We calculate the SSIM between 11-pixel windows of the real and virtual IF image tiles. The SSIM between two windows $x$ and $y$ is defined as:

$$ {\text{SSIM}}\left( {x,y} \right) = \frac{{\left( {2\mu_{x} \mu_{y} } \right)\left( {2\sigma_{xy} + c_{2} } \right)}}{{\left( {\mu_{x}^{2} + \mu_{y}^{2} + c_{1} } \right)\left( {\sigma_{x}^{2} + \sigma_{y}^{2} + c_{2} } \right)}} $$

where $\mu_{x}$ and $\mu_{y}$ are the mean intensities of $x$ and $y$, $\sigma_{xy}$ is the covariance of $x$ and $y$, $\sigma_{x}^{2}$ and $\sigma_{y}^{2}$ are the variances of $x$ and $y$, and $c_{1}$ and $c_{2}$ are stabilizing constants. The SSIM between real and virtual IF images is the SSIM averaged over their windows. Based on our observation that SSIM is sensitive to simulations of technical noise which are impossible for SHIFT models to infer (Supplementary Fig. S7), we apply Gaussian filtering to real and virtual IF images tiles before calculating SSIM using the gaussian function implemented in the Python library scikit-image^43^ with sigma set to 3. We also measured the Pearson’s correlation coefficient between images in Supplementary Fig. S7 using the pearson_r function implemented in the Python library SciPy^46^.

#### Model SSIM comparisons

The distributions of model SSIM scores were not normally distributed, as measured by the shapiro function, an implementation of the Shapiro–Wilk test in SciPy, so non-parametric test were used for comparisons of model performance. We tested for differences between SSIM scores from three models evaluated on the same sample subset using the friedmanchisquare function implemented in SciPy which computes the non-parametric paired measures Friedman test that is commonly used to measure consistency among measurements on a dataset obtained from different models. If the Friedman test rejected the null hypothesis that there was no difference in performance between three models, we then identified the models with different performance using the posthoc_nemenyi_friedman function implemented in the Python library scikit-posthocs^45^. We tested for differences between SSIM scores from two models evaluated on the same sample subset using the wilcoxon function implemented in SciPy. The significance level was set to α = 0.05.

## Supplementary information

Supplementary information1

## Data Availability

Code and data for training and inference can be found at https://gitlab.com/eburling/shift. Models are implemented in Pytorch^39^. Figure generation code is implemented in Holoviews^41^ using the Bokeh backend^42^ and Jupyter notebooks^47^.
