# Supplementary material for: SHIFT: speedy histological-to-immunofluorescent translation of a tumor signature enabled by deep learning
Source: Sci Rep. 2020 Oct 15;10:17507. doi: 10.1038/s41598-020-74500-3 (PMC7566625; doi:10.1038/s41598-020-74500-3)
Supplement: Supplementary file 1 — Supplementary information1 [file 41598_2020_74500_MOESM1_ESM.docx]

SHIFT: speedy histological-to-immunofluorescent translation of a tumor signature enabled by deep learning

Erik A. Burlingame,^1,2^ Mary McDonnell,^2^ Geoffrey F. Schau,^1,2^ Guillaume Thibault,^2^ Christian Lanciault,^3^ Terry Morgan,^3^ Brett E. Johnson,^2^ Christopher Corless,^4,5^ Joe W. Gray,^2,5,6^ Young Hwan Chang^1,2,6*^

^1^Computational Biology Program, Department of Biomedical Engineering, Oregon Health & Science University, Portland, OR, USA

^2^OHSU Center for Spatial Systems Biomedicine, Department of Biomedical Engineering, Oregon Health & Science University, Portland, OR, USA

^3^Department of Pathology, Oregon Health & Science University, Portland, OR, USA

^4^Knight Diagnostic Laboratories, Oregon Health & Science University, Portland, OR, USA

^5^Knight Cancer Institute, Oregon Health & Science University, Portland, OR, USA

^6^Brenden-Colson Center for Pancreatic Care, Oregon Health & Science University, Portland, OR, USA

^*^Corresponding author, chanyo@ohsu.edu

| Reference | Target marker | Image tiles | XY tile resolution (pixels) | Pixel resolution  (microns/pixel) | Total image area | Difference in area from current study |
| --- | --- | --- | --- | --- | --- | --- |
| Christiansen et al. [13] | DAPI | 2 | 3500×3500 | 0.32 | 78 mm^2^ | 45-fold less |
| Ounkomol et al. [14] | Lamin | 40 | 924×624 | 0.108 | 25 mm^2^ | 140-fold less |
| Current study | panCK | 12258 | 256×256 | 0.44 | 3.5 cm^2^ | − |

**Supplementary Table S1. Comparison of total image area used for training and testing of virtual staining methods.**

**
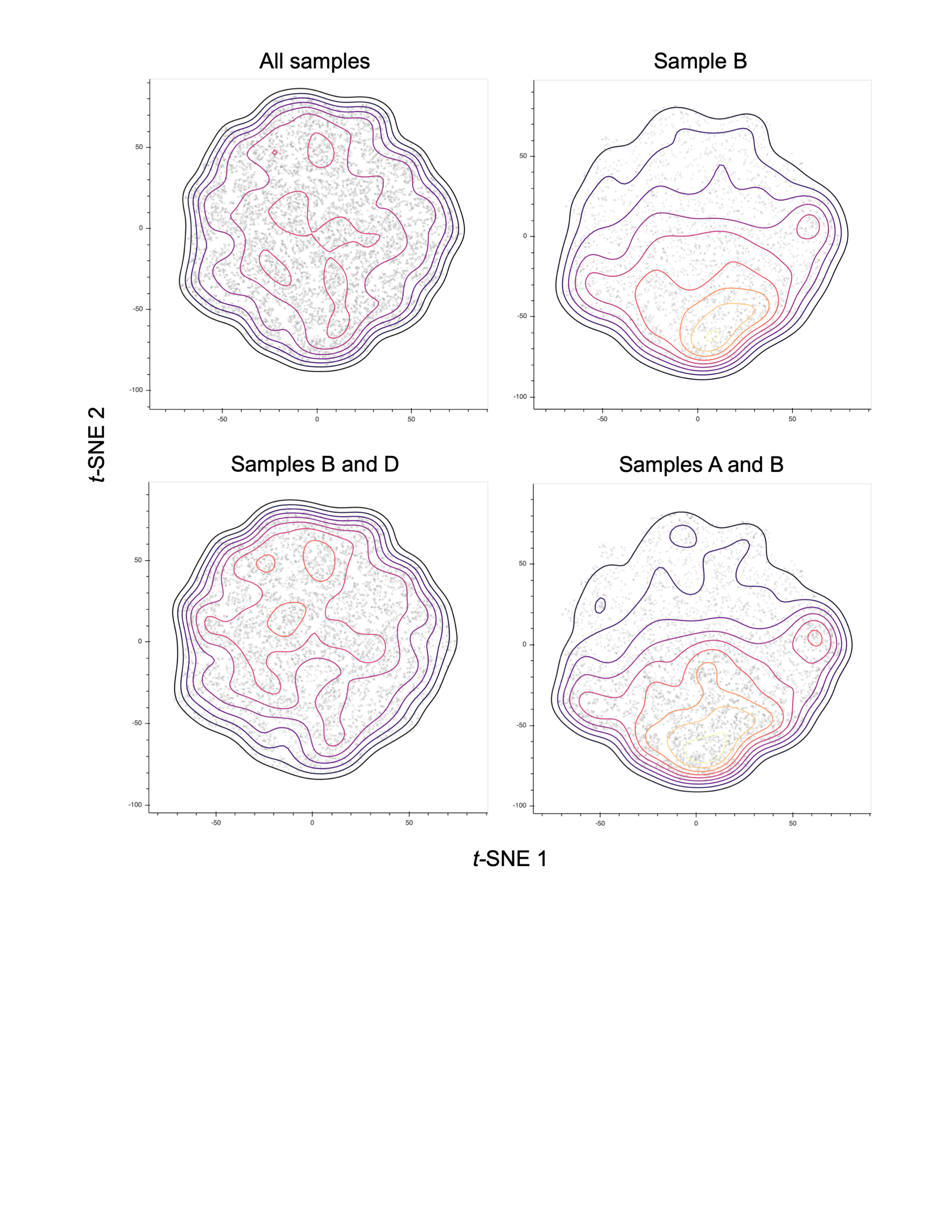
**

**Supplementary Figure S1. Related to Figure 2. H&E tile feature distributions of experiment sample combinations.** The VAE-learned 16-dimensional feature vector representations for each H&E are embedded into 2 dimensions using *t*-SNE. Each point in each plot represents a single H&E tile. Contour lines indicate point density. Sample B is the single sample that is most representative of the whole dataset. Samples B and D are the duo of samples that are most representative of the whole dataset. Samples A and B are a duo of samples that poorly represent the whole dataset.

**
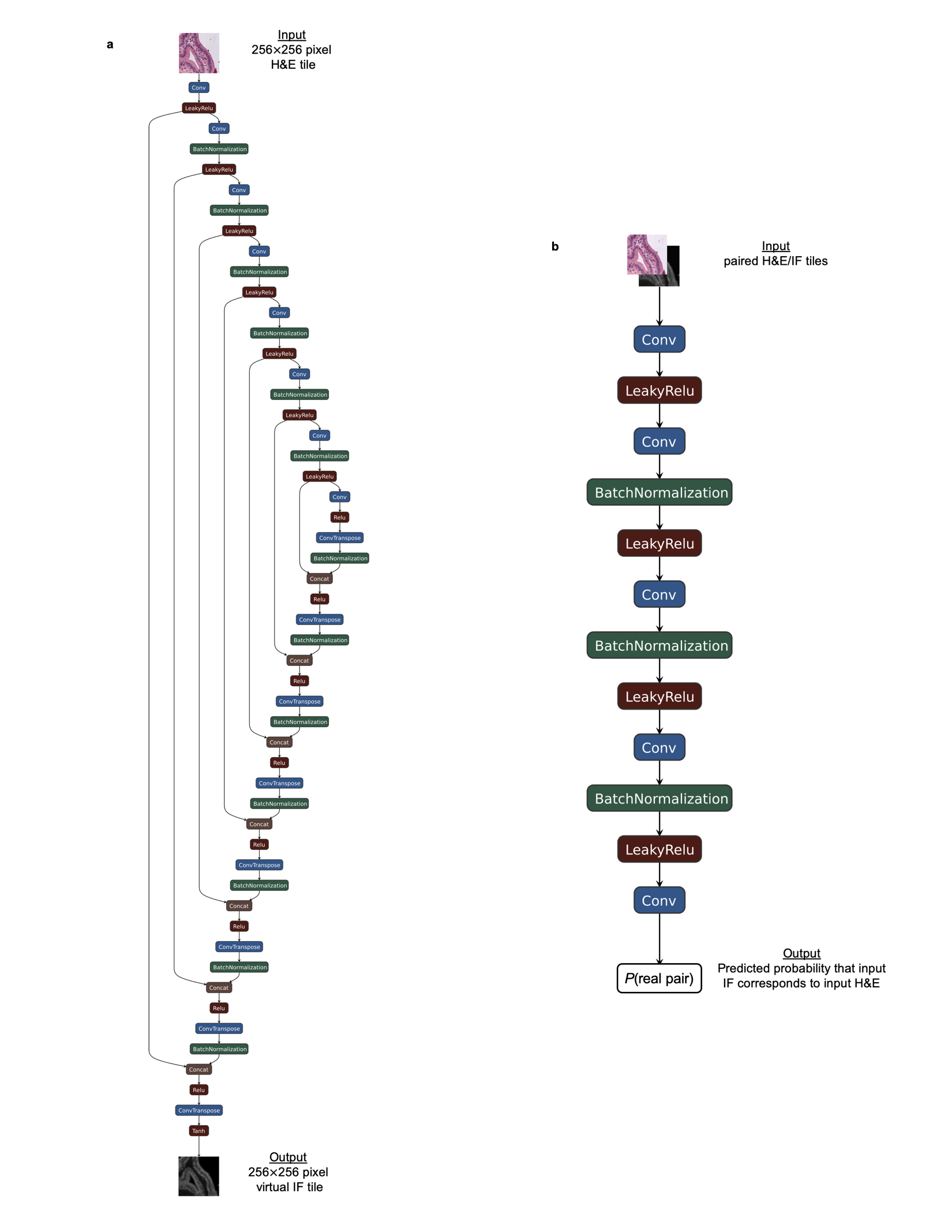
**

**Supplementary Figure S2. Related to Figure 1. Schematics of cGAN architecture used by SHIFT.** The cGAN architecture used by SHIFT is based on the *pix2pix* framework [25]. Schematics were generated with the Netron network viewer tool (https://github.com/lutzroeder/netron). Full implementation details available on GitLab (https://gitlab.com/eburling/shift).

(**a**) Architecture of generator network $G$ which is based on the U-net architecture [27].

(**b**) Architecture of discriminator network $D$. The input to $D$ is a single image of H&E and IF concatenated along the channel axis.


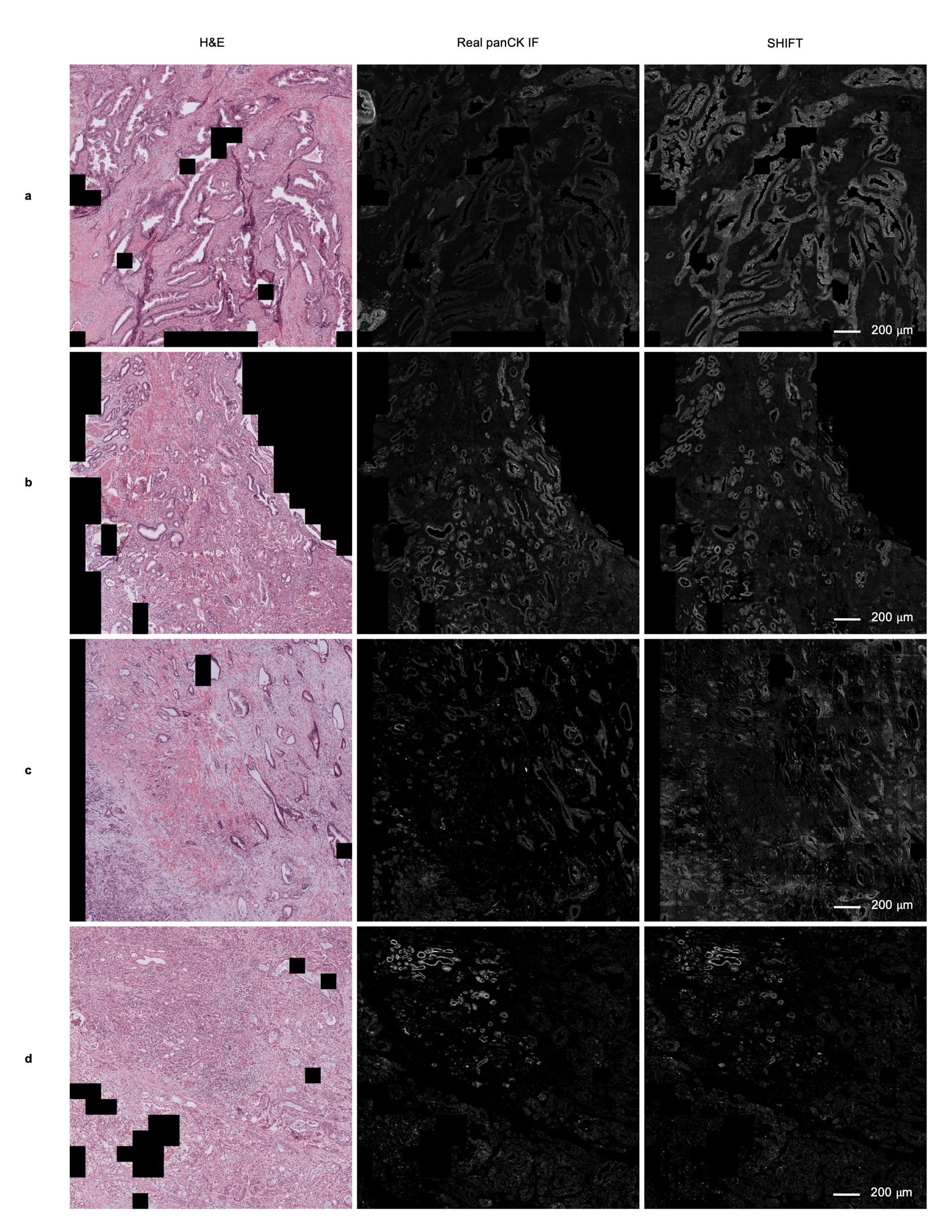


**Supplementary Figure S3. Related to Figure 2. Large-scale comparison of real and virtual panCK staining generated by SHIFT.**

SHIFT images were generated by a model trained on sample subsets B1 and D5. Results shown are from the test set. Tiles were excluded if they contained more than 50% background in the H&E representation (black tiles).

(**a**) Representative images taken from sample A. Robust staining of fibrotic vasculature in the upper- and lower-left of the real panCK image is not recapitulated in the SHIFT image because panCK+ fibrotic vasculature was not present in the model’s training set of samples B and D. Rather, the desired virtual staining of tumor epithelium is generated.

(**b**) Representative images taken from sample B.

(**c**) Representative images taken from sample C.

(**d**) Representative images taken from sample D.


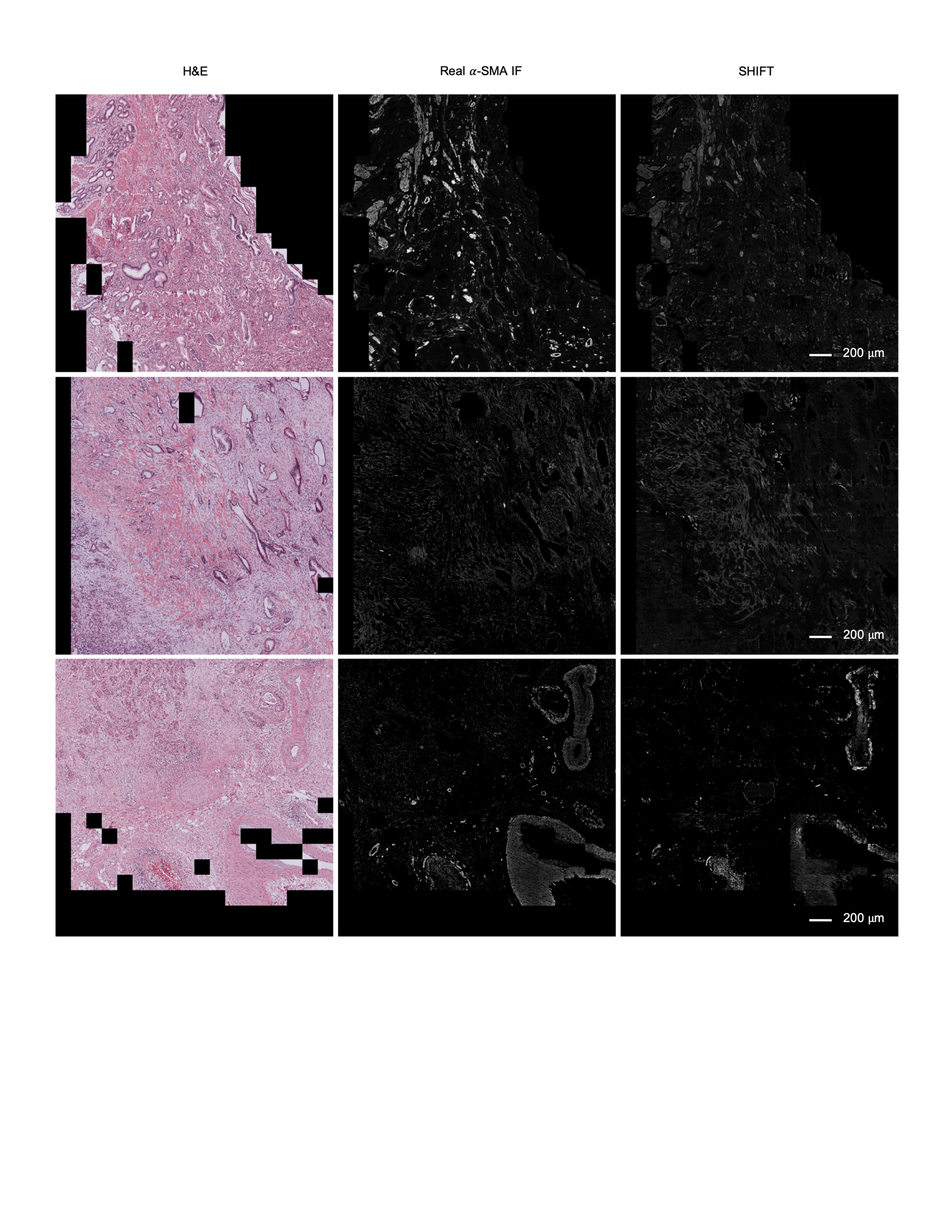


**Supplementary Figure S4. Related to Figure 2. Large-scale comparison of real and virtual α-SMA staining generated by SHIFT.**

SHIFT images were generated by a model trained on sample subsets B1 and D5. Results show are from the test set. Tiles were excluded if they contained more than 50% background in the H&E representation (black tiles). Discrepancies between the real and virtual stains suggest that our dataset is of insufficient size to optimally model the inter-sample heterogeneity of α-SMA expression.

**
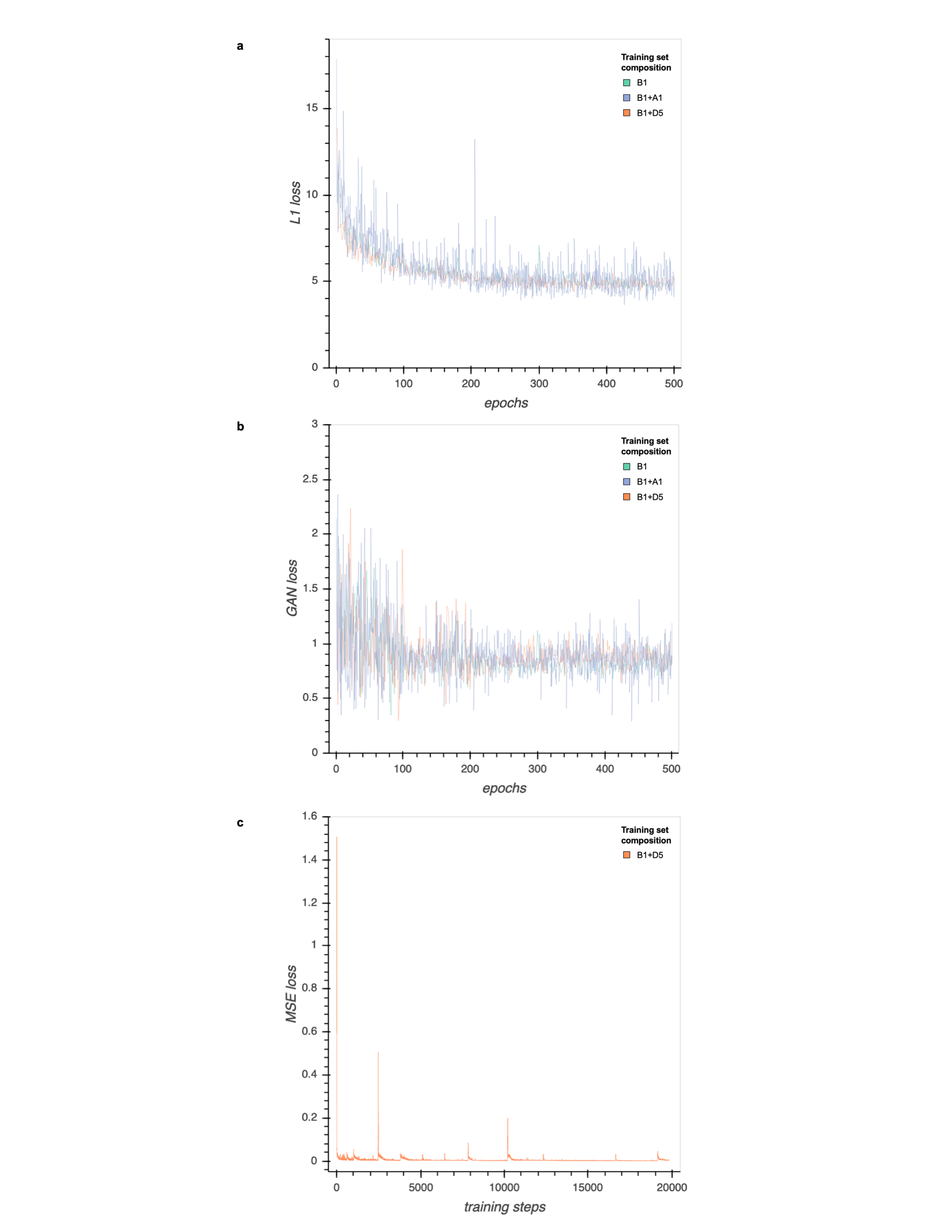
**

**Supplementary Figure S5. Related to Methods. Training losses for virtual staining models.**

(**a**) L1 training loss for SHIFT models for each training set composition.

(**b**) GAN training loss for SHIFT models for each training set composition.

(**c**) Mean squared error (MSE) training loss for Label-Free Determination (LFD) model.

**
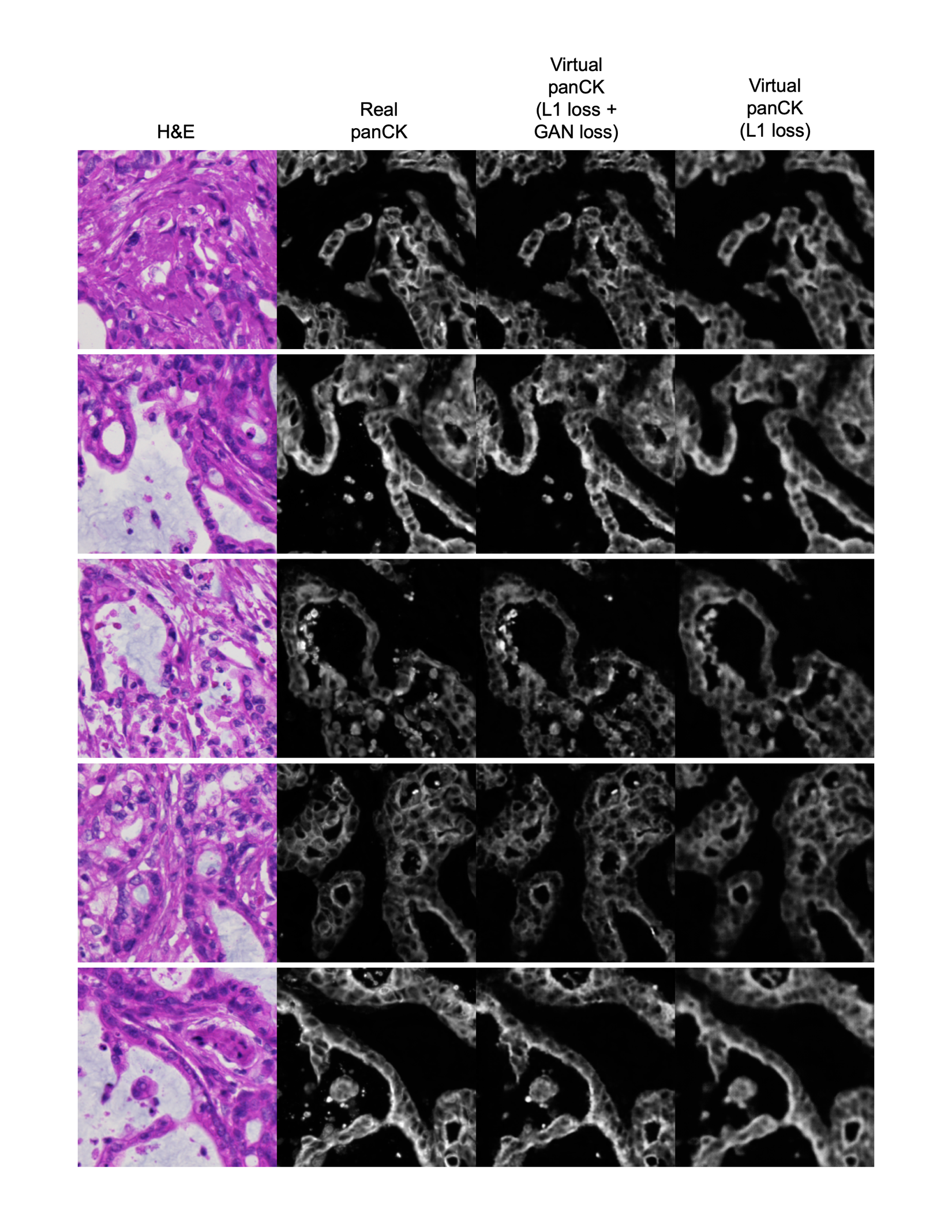
**

**Supplementary Figure S6. Related to Methods. Comparison of different training losses for models estimating panCK.** Training examples for models trained to convergence using either L1 and GAN losses, or L1 loss alone. Stain estimation for the model trained using L1 loss alone lacks high-frequency textural details of the panCK stain when compared to the model using both L1 and GAN losses.


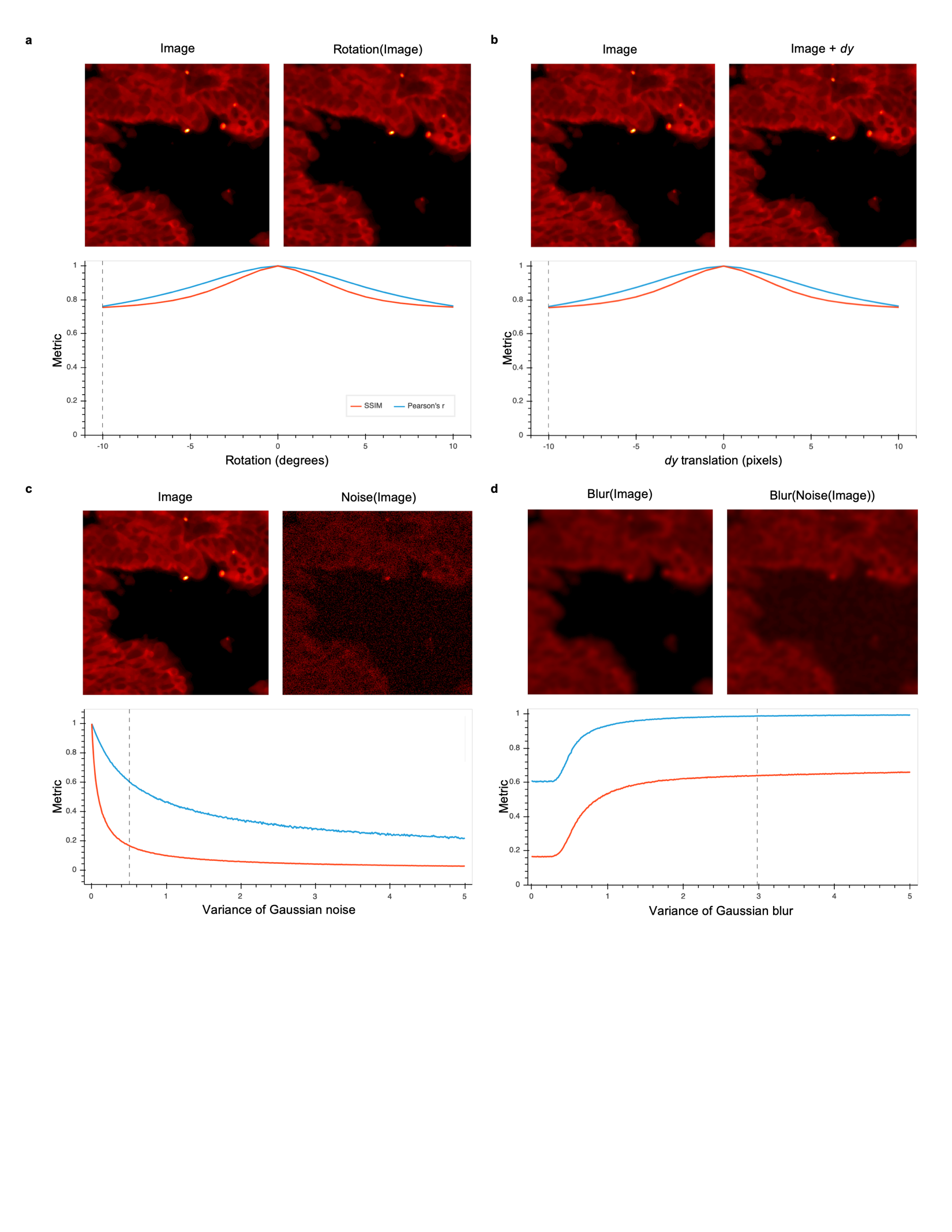
**Supplementary Figure S7. Related to Methods. Metric sensitivity to common technical perturbations.** Structural similarity (SSIM) and Pearson’s *r* are two commonly used metrics for image comparison, both having been used to make comparisons between real and generated biological images in recent related work [10,13,14]. When comparing raw real IF and raw generated IF images, a low SSIM value may be the result of sensor/technical noise in the IF procedure, which is impossible for the SHIFT model to predict based on the H&E image it is given as input. When considering a stereotypical panCK IF tile, both measures are found to be sensitive to rotation (**a**) and translation (**b**), perturbations common to image registration. Grey dotted lines indicate the parameter selected to generate each transformed image. We also observe that both measures are sensitive to simulations of technical noise (**c**). By applying a Gaussian filter with variance (sigma) set to 3, we recover the SSIM between real and perturbed IF images without sacrificing global image details (**d**).
